# Identification and verification of diagnostic biomarkers based on mitochondria-related genes related to immune microenvironment for preeclampsia using machine learning algorithms

**DOI:** 10.3389/fimmu.2023.1304165

**Published:** 2024-01-08

**Authors:** Pu Huang, Yuchun Song, Yu Yang, Feiyue Bai, Na Li, Dan Liu, Chunfang Li, Xuelan Li, Wenli Gou, Lu Zong

**Affiliations:** ^1^ Department of Obstetrics & Gynecology, the First Affiliated Hospital of Xi’an Jiaotong University, Xian, Shaanxi, China; ^2^ Department of Gynecology and Obstetrics, Yantai Affiliated Hospital of Binzhou Medical University, Yantai, Shandong, China

**Keywords:** preeclampsia, mitochondria-related genes, immune microenvironment, machine learning, diagnostic model

## Abstract

Preeclampsia is one of the leading causes of maternal and fetal morbidity and mortality worldwide. Preeclampsia is linked to mitochondrial dysfunction as a contributing factor in its progression. This study aimed to develop a novel diagnostic model based on mitochondria-related genes(MRGs) for preeclampsia using machine learning and further investigate the association of the MRGs and immune infiltration landscape in preeclampsia. In this research, we analyzed GSE75010 database and screened 552 DE-MRGs between preeclampsia samples and normal samples. Enrichment assays indicated that 552 DE-MRGs were mainly related to energy metabolism pathway and several different diseases. Then, we performed LASSO and SVM-RFE and identified three critical diagnostic genes for preeclampsia, including CPOX, DEGS1 and SH3BP5. In addition, we developed a novel diagnostic model using the above three genes and its diagnostic value was confirmed in GSE44711, GSE75010 datasets and our cohorts. Importantly, the results of RT-PCR confirmed the expressions of CPOX, DEGS1 and SH3BP5 were distinctly increased in preeclampsia samples compared with normal samples. The results of the CIBERSORT algorithm revealed a striking dissimilarity between the immune cells found in preeclampsia samples and those found in normal samples. In addition, we found that the levels of SH3BP5 were closely associated with several immune cells, highlighting its potential involved in immune microenvironment of preeclampsia. Overall, this study has provided a novel diagnostic model and diagnostic genes for preeclampsia while also revealing the association between MRGs and immune infiltration. These findings offer valuable insights for further research and treatment of preeclampsia.

## Introduction

Preeclampsia represents a pregnancy-associated syndrome typified by elevated blood pressure and the presence of protein in the urine. This condition initiates a spectrum of pathophysiological mechanisms, encompassing endothelial dysfunction, systemic inflammation, and compromised implantation processes (1, 2). Approximately 5-8% of pregnancies are affected by preeclampsia worldwide. While the overall frequency is lower in industrialized countries, it is higher in some parts of the world that are still developing (3, 4). Primiparity (first pregnancy), advanced or young maternal age, hypertension, renal disease, diabetes, obesity, multiple gestations (twins or triplets), and some hereditary variables all enhance the likelihood of developing preeclampsia. Although preeclampsia has been documented all across the world, its incidence varies considerably depending on location (5, 6). These variations in geographic distribution may be influenced by factors such as diet, lifestyle, and genetic predisposition. Preeclampsia’s pathogenesis remains a mystery, despite decades of research into its possible causes. Only timely termination of pregnancy, along with hypertension control and close monitoring of mother and baby, has been shown to be successful in the treatment of preeclampsia (7–9). Therefore, reducing the risk of unfavorable maternal and fetal outcomes requires early discovery, correct diagnosis, and effective care of individuals with preeclampsia.

Mitochondria are an important component of the cellular machinery. They generate cellular energy in the form of adenosine triphosphate (ATP), hence they are often called “the powerhouses of the cell.” (10). The outer membrane of a mitochondrial cell surrounds the inner membrane, creating a double-membrane structure. The inner membrane is highly efficient in producing energy due to its many folds, or cristae, which enhance the membrane’s surface area (11). Mitochondrial DNA (mtDNA) is contained within mitochondria, along with several proteins involved in the process of producing energy (12). Mitochondria are essential for cellular energy metabolism. However, there is evidence from a subset of research suggesting that mitochondrial activity is impaired and energy metabolism is disrupted in patients with preeclampsia. This could lead to a shortage of cellular energy, which in turn could harm a pregnant woman’s organs. Oxidative stress has also been linked to mitochondria (13, 14). To put it simply, oxidative stress is a process that can cause damage to cells and tissues. Some studies have found that preeclampsia patients have elevated levels of oxidative stress, which can lead to cell death and inflammatory reactions. Oxidative stress has been linked to mitochondrial malfunction (15, 16). Additionally, mitochondria have their own DNA, separate from that of the cell nucleus. Because mitochondria produce so many free radicals during the energy generating process, mitochondrial DNA is especially susceptible to oxidative damage and other sorts of injury (17). Some studies suggest that in preeclampsia patients, damage to mitochondrial DNA may be elevated, which could be related to the pathogenesis of preeclampsia (18–20). More importantly, some mitochondria-related genes have been reported to exhibit a dysregulated level in preeclampsia (21–23). Thus, certain crucial mitochondrial-related genes may emerge as novel diagnostic biomarkers for preeclampsia. In addition, understanding the intricate relationship between mitochondria and preeclampsia is essential for advancing research and potential treatments.

Machine learning is a branch of artificial intelligence (AI) that focuses on researching how to use computer systems to automate learning and improvement of tasks without explicit programming (24). Its core idea is to enable computer systems to make predictions or decisions by discovering patterns and regularities from data. Image recognition, NLP, medical diagnosis, financial forecasting, and autonomous cars are just few of the many areas where machine learning has found success. Machine learning has becoming increasingly important in the medical industry, especially in genomics and illness diagnostics (25, 26). Transcriptomics is a method for analyzing the abundance of gene expression data collected from within cells. Many scientists are now combining machine learning with transcriptomics sequencing to pinpoint important diagnostic genes. Transcriptomic data can be used by machine learning algorithms to identify cell kinds, disease states, and physiological circumstances. Classification algorithms can be used, for instance, to determine which genes show statistically significant expression differences between people with cancer and those without the disease (27, 28). New gene markers or biomarkers, useful for early disease diagnosis, prognosis prediction, and treatment selection, can be found with the help of machine learning-based techniques. These algorithms can examine massive amounts of data to pinpoint which genes are connected with a disease’s development and response to treatment. In conclusion, using machine learning in transcriptome sequencing and illness diagnosis gene study helps speed up medical research and improve diagnostic accuracy. It assists scientists in discovering novel gene markers, comprehending disease mechanisms, predicting patients’ disease risks, and providing more precise treatment strategies for individuals. Despite the widespread use of machine learning in medical research, its application for identifying novel biomarkers in preeclampsia remains underexplored. This gap in the literature highlights the need for research exploring the potential of machine learning in uncovering biomarkers specific to preeclampsia. In this context, our study aims to bridge this gap by leveraging machine learning techniques in transcriptome sequencing to identify novel biomarkers based on mitochondrial-related genes(MRGs) for preeclampsia.

Up to date, there has been no studies to utilize machine learning for the analysis of MRGs, to screen for pivotal diagnostic genes, and to construct a diagnostic model. In this study, we aimed to developed a novel diagnostic model using MRGs based on Machine learning. In addition, we further analyzed the association between the critical genes and immune infiltration. In summary, our research not only presented a pioneering diagnostic model for preeclampsia based on MRGs and machine learning but also provided crucial insights into the molecular intricacies underpinning the relationship between MRGs and preeclampsia. Moreover, our findings regarding the association with immune infiltration had potential implications for advancing therapeutic strategies, opening avenues for targeted interventions and personalized treatment approaches in the realm of preeclampsia management.

## Materials and methods

### Serum samples

In this study, 12 preeclampsia patients aged 25 to 40 and 12 healthy donors aged 23 to 40 were enrolled from the First Affiliated Hospital of Xi’an Jiaotong University. In addition, we collected blood samples from 12 preeclampsia patients and 12 participants, all at gestational weeks 20 to 25. The patients had not received any therapy for preeclampsia before sample collection. All participants provided written informed consent. This study was approved by the Ethics Committee of the First Affiliated Hospital of Xi’an Jiaotong University. The blood samples were centrifuged at 1,500 × g in a refrigerated centrifuge (Eppendorf Centrifuge 5430R; Eppendorf, Hamburg, Germany) for 12 min. This process effectively separated the serum from the cellular components of the blood. The centrifugation step was conducted under standard laboratory conditions to ensure reproducibility and consistency in the experimental procedure. The blood samples were stored at -80 °C until use.

### Quantitative RT-PCR

TRIzol LS reagent (#10296010, Invitrogen) was used to extract the total RNA from each sample. HiFiScript cDNA Synthesis Kit (CWBIO, China) was used for reverse transcription of total RNA to produce cDNA. gene expression levels were assessed through quantitative real-time polymerase chain reaction (qRT-PCR) utilizing the Ultra SYBR Mixture, manufactured by Vazyme in China. The RT-qPCR was performed with cycling conditions as follows: 15 min. 50°C, 2 min. 95°C, (15 s 95°C, 32 s 60°C), 45 cycles. To ensure accuracy, the relative gene expression levels were standardized against the expression levels of GAPDH and were calculated utilizing the 2^(-ΔΔCt) method. The primers used in this study were shown below: for CPOX, 5’- GCTGGGGTGAGCATTTCTGTT-3’ (forward), 5’- GCATGAGGATTCTTGGGGTGG-3’(reverse); for DEGS1, 5’- GAGATCCTGGCAAAGTATCCAGA-3’ (forward), 5’- CAAACGCATAGGCCCCAAA-3’(reverse); SH3BP5, 5’- GAGCGAGCTGGTGCATAAGG-3’ (forward), 5’- TGGACTTGTTGATGGCTCTCT-3’(reverse); GAPDH, 5’- ACAACTTTGGTATCGTGGAAGG-3’ (forward), 5’- GCCATCACGCCACAGTTTC -3’(reverse).

### Data source

Gene Expression Omnibus (GEO) is an online biological information resource maintained by the National Center for Biotechnology Information (NCBI) in the United States. GEO is designed to store and share gene expression data, providing a publicly accessible platform for researchers, biologists, and bioinformaticians to store, retrieve, analyze, and share large-scale gene expression and functional genomics data. GEO is a global database that allows researchers to upload their own gene expression data to the platform. This facilitates data sharing and accessibility, thereby promoting scientific collaboration and data reuse. GEO offers a range of tools and resources for data analysis and visualization, helping researchers interpret and unearth biological information within the data. These tools assist researchers in identifying gene expression patterns, biological pathways, and genes associated with diseases. Two RNA-sequence files of preeclampsia samples and normal samples were extracted from the GEO datasets, including GSE44711 and GSE75010 datasets. Furthermore, we screened MRGs with a count of 1513 from MSigDB (https://www.gsea-msigdb.org/gsea/msigdb).

### Differential expression analysis

From the GSE75010 database, we sourced the expression profiles for 1513 MRGs present in preeclampsia samples and normal samples. Then, using the R platform, we employed the student’s t-test to identify any MRGs exhibiting differential expression between the two sample sets. If the p-value was less than 0.05, it was considered to be statistically significant.

### Functional enrichment analyses

Gene Ontology(GO) analysis, is a bioinformatics tool and method used to study and interpret the biological functions and processes within large-scale genomics data. GO analysis relies on the Gene Ontology database, which is a maintained and regularly updated standardized biological terminology repository used to describe the functions, cellular components, and biological processes of genes and proteins. GO analysis was performed on DE-MRGs using EnrichGO function in the R package “clusterProfiler”. “clusterProfiler” is a commonly used R package in the field of bioinformatics, employed for functional enrichment analysis and visualization. It is typically utilized to interpret high-throughput experimental data, such as gene expression or proteomics data, to identify functional features of a set of genes or proteins (29). GO analysis helps reveal the functions of the identified genes in terms of cellular molecular functions, biological processes, and cellular components. Kyoto Encyclopedia of Genes and Genomes Analysis(KEGG) analysis is a bioinformatics method used to study and interpret biological pathways and metabolic pathways within genomics data. It relies on the KEGG database, which provides detailed information about biological pathways, metabolic pathways, and associated genes, assisting researchers in gaining a deeper understanding of the roles and interactions of genes and proteins in biological processes. The KEGG database contains information about various biological pathways and metabolic pathways, spanning multiple species. These pathways describe the interactions and signaling between different biological molecules within an organism, including genes, proteins, metabolites, and more. The KEGG database is a crucial resource for comprehending the regulation mechanisms of gene functions and biological processes. KEGG analysis was performed using the EnrichKEGG function of the R package “clusterProfiler”. KEGG analysis is introduced to explain the involvement of these genes in the metabolism and signaling pathways within biological systems. Gene Set Enrichment Analysis(GSEA) is a bioinformatics method used to analyze and interpret the enrichment of biological processes and pathways in gene expression data. Its primary objective is to determine whether a set of genes is enriched in specific biological pathways or functional collections, thereby aiding researchers in understanding the biological significance of gene expression data. The R “clusterProfiler” package’s gseGO, gseKEGG, and gsePathway functions were used to conduct the GSEA. GSEA possesses significant advantages in studying the overall trends of gene sets, allowing for a more comprehensive understanding of the functions of these genes within specific diseases. Enrichment analyses utilizing Disease Ontology (DO) were executed on DE-MRGs through the utilization of the “clusterProfiler” and “DOSE” packages within the R programming environment. The threshold conditions were a significance level of p-value less than 0.05 and an adjusted p-value less than 0.05.

### Identification of optimal diagnostic gene biomarkers for preeclampsia

The least absolute shrinkage and selection operator (LASSO) is a widely used regularization technique in machine learning and statistics (30). It was introduced by Robert Tibshirani in 1996 and is primarily used for linear regression, though it has been extended to other models as well. The LASSO algorithm is designed to address the issue of overfitting and perform feature selection by adding a penalty term to the linear regression cost function. The glmnet package was employed to implement the LASSO algorithm, which played a pivotal role in dimensionality reduction of the dataset. Specifically, it was used to identify the DE-MRGs when comparing preeclampsia patients with normal samples. Through this feature selection process, the LASSO algorithm aided in pinpointing key gene biomarkers associated with preeclampsia. Support vector machine-recursive feature elimination (SVM-RFE) is a feature selection technique used in machine learning (31, 32). It combines the principles of Support Vector Machines (SVM) and recursive feature elimination (RFE) to identify and rank the most important features in a dataset. The SVM is a robust supervised learning technique for classification and regression. The method finds a hyperplane that maximally splits data points into their respective classes. Meanwhile, an SVM-RFE model was constructed using an SVM package, and its performance was compared based on the average misclassification rates observed in 10-fold cross-validation. Additionally, the identification of optimal gene biomarkers for preeclampsia involved the convergence of biomarkers selected by both algorithms. The diagnostic potential of these optimal gene biomarkers was evaluated through the calculation of receiver operating characteristic (ROC) curves. Furthermore, to classify samples in the GSE75010 database, a logistic regression model was constructed using marker genes.

### Immune infiltration analysis

CIBERSORT is a computational algorithm and tool used in the field of bioinformatics and genomics to estimate the composition of cell types within a mixed cell population, typically based on gene expression data (33). The name CIBERSORT stands for “Cell-type Identification By Estimating Relative Subsets of RNA Transcripts.” This method is particularly valuable in the analysis of bulk gene expression data, where the goal is to deconvolute the gene expression profiles of different cell types within a tissue or sample. CIBERSORT is widely applied in the study of tumor microenvironments, immune cell infiltration, and related fields (34, 35). Through CIBERSORT, researchers can gain a more comprehensive understanding of the presence and relative proportions of different cell types in the samples, providing robust support for further biological investigations. Using CIBERSORT, we calculated the percentages of several immune cell types present in low-expression and high-expression cohorts. The sum of the predicted values for the various immune cell types gives each sample a total score of one.

### Statistical analysis

R 4.1.0 was used for all data manipulation, statistical analysis, and visualization. The statistical significance of differences between two groups was determined using either the Student’s t-test or the Wilcoxon rank-sum test. All tests were two-tailed, and a significance level of P 0.05 was considered to have been reached.

## Results

### Identification of DE-MRGs in preeclampsia patients

**Figure 1:**
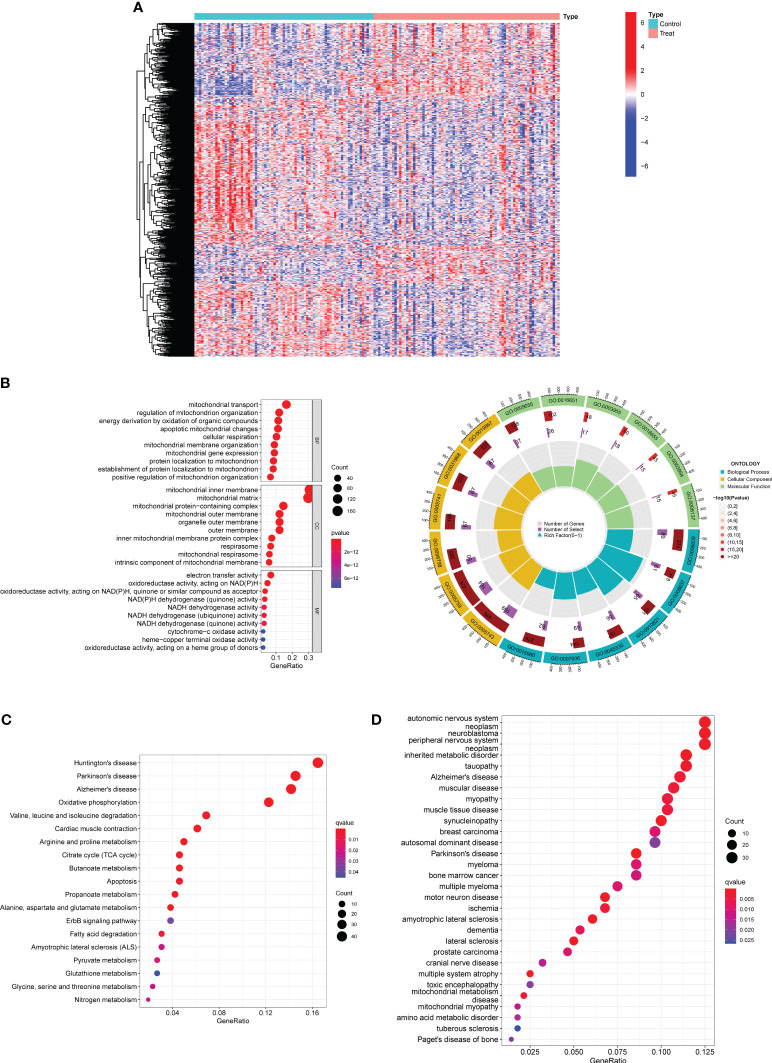
Identification of DE-MRGs in preeclampsia samples and Functional Correlation Analysis. **(A)** Violin plots were employed to visually represent the differential expression patterns of DE-MRGs between samples derived from individuals with preeclampsia and those from individuals with normal pregnancy. **(B)** GO enrichment analyses were conducted to unveil the functional implications of the identified DE-MRGs. **(C)** Enrichment analyses based on the KEGG were employed to elucidate the pathway-level significance of the identified genes. **(D)** Enrichment analyses utilizing the Disease Ontology (DO) were performed to ascertain the disease-relevant associations of the identified genes.

Firstly, a retrospective analysis was performed on the data obtained from a total of 80 preeclampsia cases and 77 normal samples from the GSE75010 datasets. The DE-MRGs of the metadata were analyzed using the limma package. A total of 552 DE-MRGs were obtained: 188 genes were significantly upregulated and 364 genes were significantly downregulated (**Figure 1A**).

### Functional correlation analysis

Then, we performed Functional Correlation Analysis to explore the possible function of 552 DE-MRGs in preeclampsia progression. The results of GO analysis revealed that 552 DE-MRGs were mainly enriched in mitochondrial transport, regulation of mitochondrion organization, energy derivation by oxidation of organic compounds, mitochondrial inner membrane, mitochondrial matrix, mitochondrial protein-containing complex, electron transfer activity, oxidoreductase activity, acting on NAD(P)H and NAD(P)H dehydrogenase (quinone) activity (**Figure 1B**). Then, our group carried out KEGG assays and observed that 552 DE-MRGs were mainly associated with Huntington’s disease, Parkinson’s disease, Alzheimer’s disease, Oxidative phosphorylation and Valine, leucine and isoleucine degradation (**Figure 1C**). Moreover, the results of DO analysis revealed that 552 DE-MRGs were mainly related to autonomic nervous system neoplasm, neuroblastoma, peripheral nervous system neoplasm, inherited metabolic disorder, tauopathy, Alzheimer’s disease and muscular disease (**Figure 1D**).

### Identification of diagnostic genes using LASSO

**Figure 2:**
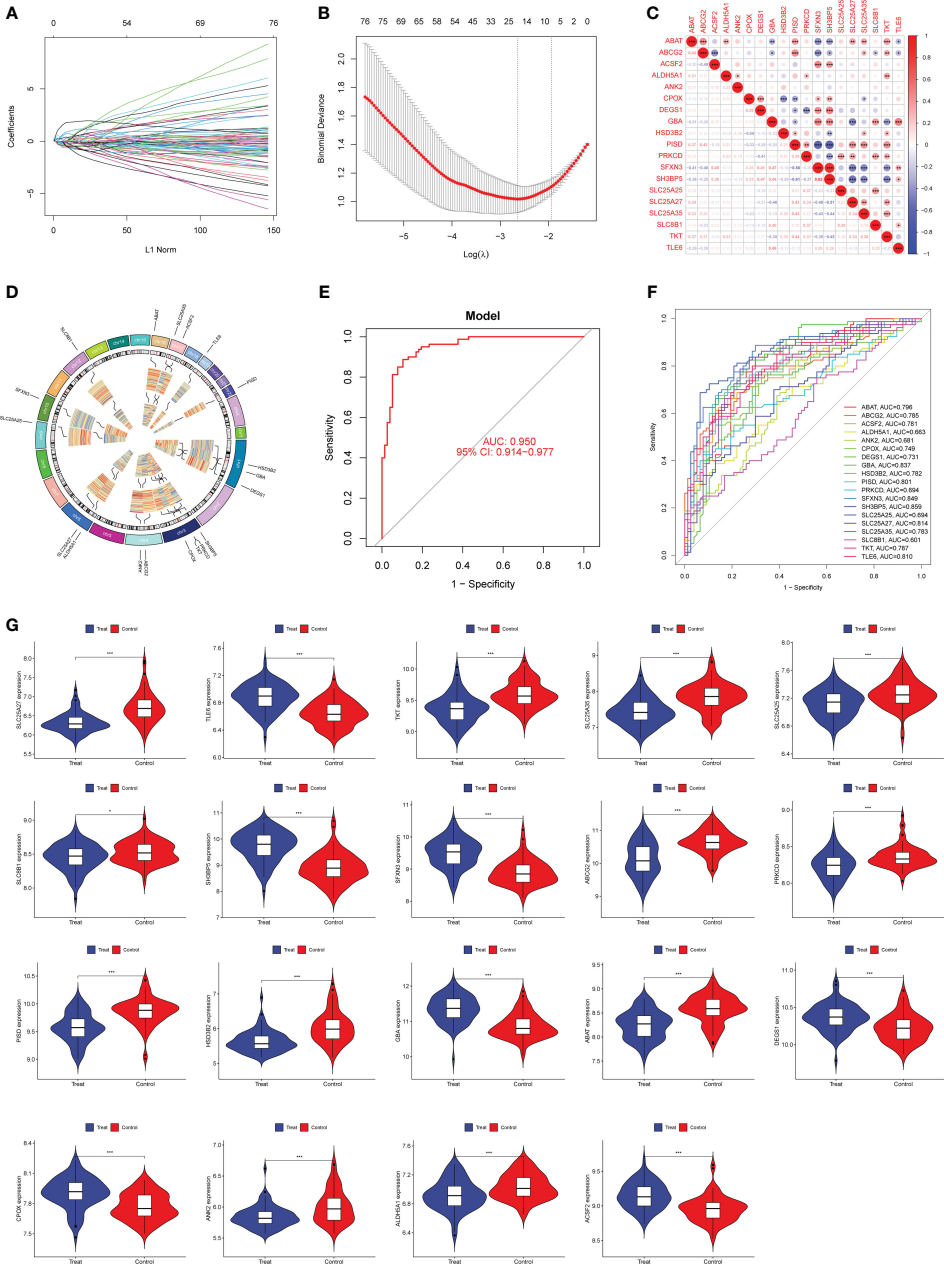
Nineteen DE-MRGs were identified as diagnostic genes for preeclampsia using LASSO. **(A, B)** Preeclampsia-related features were selected using the LASSO logistic regression algorithm, with penalty parameter adjustment achieved via a stringent 10-fold cross-validation process. **(C)** The examination of interrelationships among the 19 diagnostic genes involved a comprehensive correlation analysis. **(D)** Chromosomal locations of 19 genes. **(E)** Preeclampsia AUC determined using a logistic regression model. **(F)** ROC curves were generated to assess the performance of the 19 marker genes. **(G)** The expression profiles of the 19 genes in samples from individuals with preeclampsia. *p<0.05, **p<0.01, ***p<0.001.

Our aim was to estimate the diagnostic power of DE-MRGs by considering the disparities between preeclamptic and healthy populations. Next, we used a machine learning algorithm called LASSO on the GSE75010 dataset to screen the significant DE-MRGs in order to differentiate preeclampsia from normal samples. The LASSO logistic regression approach was used to extract 19 characteristics associated with preeclampsia, with the penalty parameters tuned using 10-fold cross-validation (**Figures 2A, B**). The results of correlation analysis of 19 genes were shown in **Figure 2C** and we observed that many of these genes exhibit significant positive or negative correlations. In addition, Chromosomal locations of candidate 19 genes were shown in **Figure 2D**. We built a logistic regression model using the R package glm based on these 19 marker genes, and the resulting ROC curves showed that the model successfully distinguished between normal and preeclampsia samples (AUC = 0.950) (**Figure 2E**). Furthermore, ROC curves were developed for the 19 marker genes to highlight their efficacy in discriminating preeclampsia from normal data. **Figure 2F** demonstrated that the AUC was higher than 0.6 for all genes and their expression pattern was shown in **Figure 2G**.

### Identification of diagnostic genes using SVM-RFE

**Figure 3:**
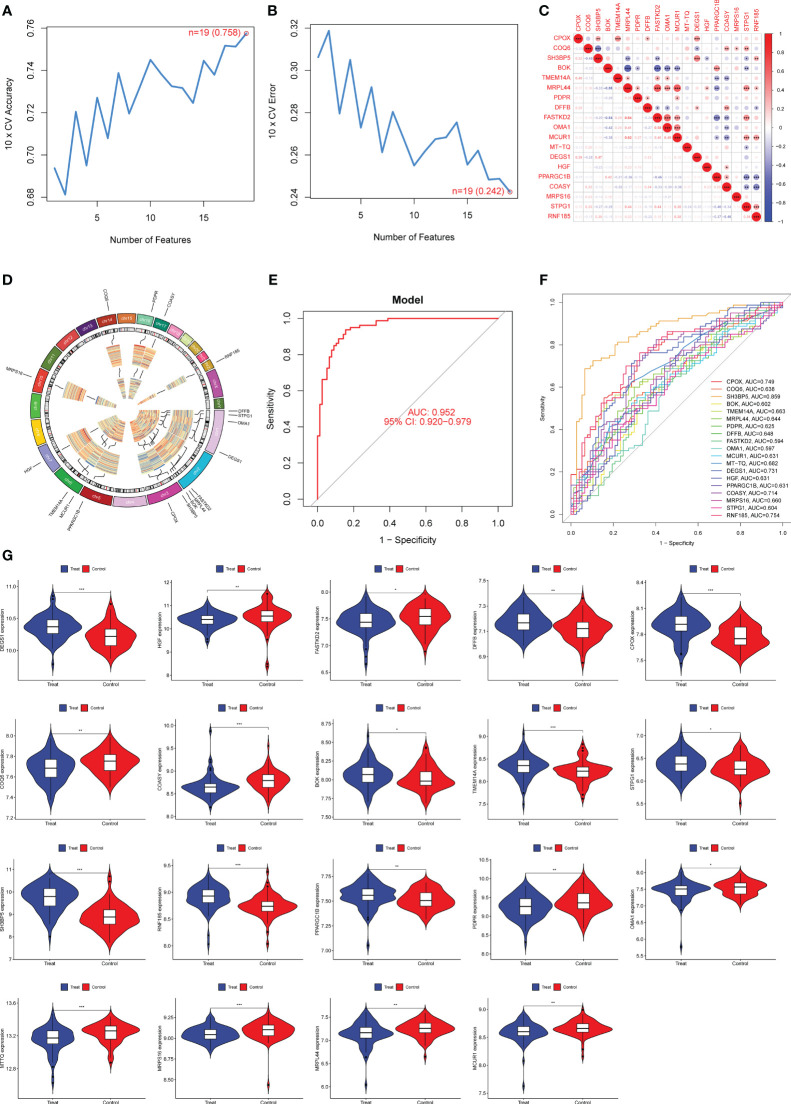
Nineteen DE-MRGs were identified as diagnostic genes for preeclampsia using SVM-RFE. **(A, B)** To narrow down the DE-MRGs and find the best set of feature genes, the SVM-RFE algorithm was used. **(C)** The correlation analysis of 19 diagnostic genes. **(D)** Chromosomal locations of 19 genes. **(E)** Preeclampsia AUC determined using a logistic regression model. **(F)** ROC curves for the 19 marker genes. **(G)** The expression pattern of 19 genes in preeclampsia samples. *p<0.05, **p<0.01, ***p<0.001.

Next, we used the SVM-RFE technique to narrow down the 552 DE-MRGs and zero in on the best set of feature genes. The best feature genes, totaling 19, were then determined (**Figures 3A, B**). The results of correlation analysis of 19 genes were shown in **Figure 3C**. In addition, chromosomal locations of candidate 19 genes were shown in **Figure 3D**. We built a logistic regression model using the R package glm based on these 19 marker genes, and the resulting ROC curves showed that the model successfully distinguished between normal and preeclampsia samples (AUC = 0.952) (**Figure 3E**). Furthermore, ROC curves were developed for the 19 marker genes to highlight their efficacy in discriminating preeclampsia from normal data. **Figure 3F** displayed that the AUC was higher than 0.6 for the vast majority of genes and their expression pattern was shown in **Figure 3G**.

### A novel diagnostic mode was developed using both LASSO and SVM-RFE

**Figure 4:**
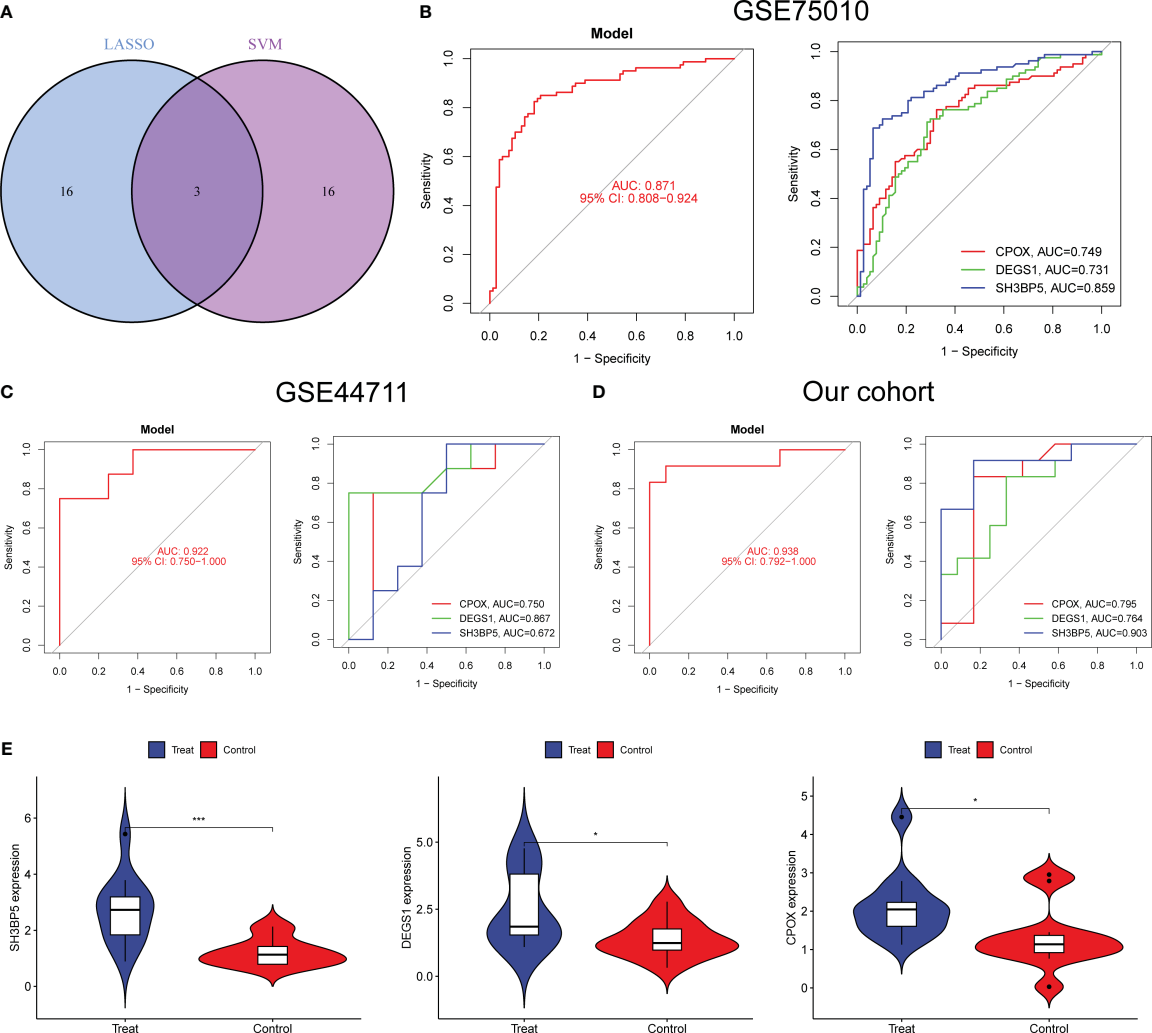
A novel diagnostic model was developed using LASSO and SVM-RFE. **(A)** The marker genes obtained from the LASSO and SVM-RFE models. **(B–D)** The diagnostic value of the new model was confirmed in G GSE75010, GSE44711 datasets and our cohort. **(E)** The expressing pattern of CPOX, DEGS1 and SH3BP5 in preeclampsia samples and normal samples from our cohort. *p<0.05, ***p<0.001.

To developed a better diagnostic model using DE-MRGs, we used a Venn diagram to obtain the intersection of LASSO and SVM-RFE, resulting in the identification of three overlapping genes, including CPOX, DEGS1 and SH3BP5 (**Figure 4A**). Next, we established a novel diagnostic model by combining CPOX, DEGS1, and SH3BP5. The ROC analysis showed that the 3-marker gene-based logistic regression model successfully distinguished between normal and preeclampsia samples (AUC = 0.871) (**Figure 4B**). In addition, the AUC for all three genes was greater than 0.7. Moreover, the diagnostic value of the new model was further confirmed in GSE44711 (**Figure 4C**) and our cohort (**Figure 4D**). Compared to normal samples, preeclampsia samples showed markedly elevated expression of CPOX, DEGS1, and SH3BP5, as determined by RT-PCR (**Figure 4E**).

### Relationship between CPOX, DEGS1 and SH3BP5 with the proportion of infiltrating immune cells

**Figure 5:**
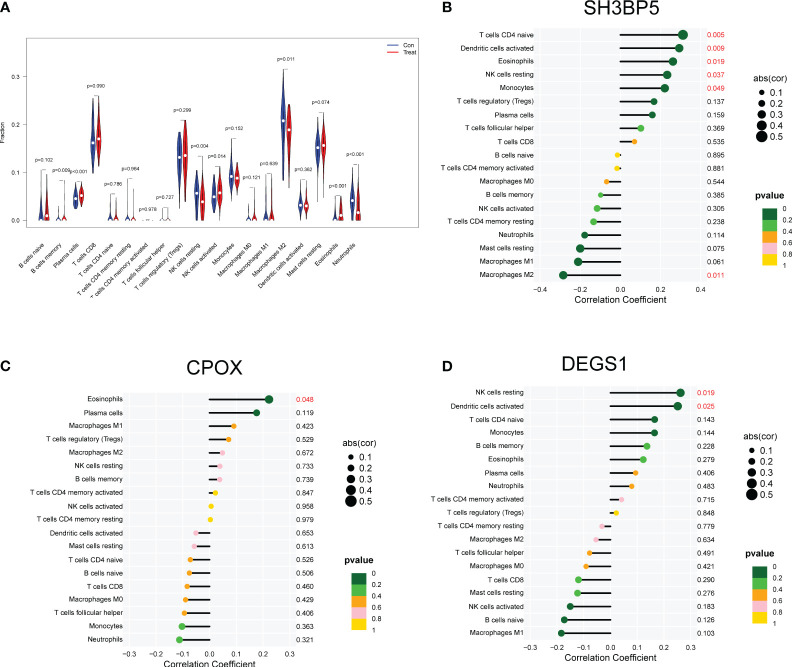
An in-depth analysis of the immune landscape to gain a comprehensive understanding of how the immune system performs and interacts within preeclampsia. **(A)** The CIBERSORT algorithm was employed to investigate disparities in the immune microenvironment between individuals with preeclampsia and a control group comprising normal samples. **(B–D)** Correlation between SH3BP5, CPOX and DEGS1 and infiltrating immune cells in preeclampsia samples.

Using the CIBERSORT method, we confirmed that there was a correlation between the expression of CPOX, DEGS1, and SH3BP5, as well as the immune system. Compared to control samples, preeclampsia samples showed a dysregulated amount of several immune cells, such as B cells memory, Plasma cells, NK cells resting, NK cells activated, Macrophages M2, Eosinophils and Neutrophils (**Figure 5A**). Then, we found that the level of SH3BP5 was positively associated with the levels of T cells CD4 naïve(cor=0.311, p = 0.005), Dendritic cells activated(cor= 0.293, p= 0.009), Eosinophils(cor= 0.261, p= 0.019), NK cells resting(cor= 0.233, p= 0.037) and Monocytes(cor= 0.221, p= 0.049), while negatively associated with Macrophages M2(cor= -0.284, p= 0.0101) (**Figure 5B**). The levels of CPOX were positively associated with the levels of Eosinophils(cor= 0.222, p= 0.048) (**Figure 5C**). In addition, The levels of DEGS1 were positively NK cells resting(cor= 0.261, p= 0.019) and Dendritic cells activated (cor= 0.251, p= 0.025) (**Figure 5D**).

## Discussion

Preeclampsia, as a severe complication posing a threat to the health of both pregnant women and fetuses, has long been a subject of significant concern (36, 37). The prognosis of preeclampsia involves two crucial aspects: the mother and the fetus. Treatment and control in a timely manner can save the life of the mother. Organ malfunction and potentially fatal consequences can result from poorly treated preeclampsia (38). Women who have had preeclampsia during pregnancy may also be more likely to have cardiovascular disease and hypertension later in life. Fetal growth limitation, intrauterine distress, premature birth, and even stillbirth have all been linked to preeclampsia because of its effect on the fetus’s blood supply and oxygen delivery (15, 39). Therefore, eclampsia poses significant challenges to the fetal prognosis as well. Early diagnosis of eclampsia is of utmost importance because it provides an opportunity for intervention and reduces the risks for both the mother and the fetus. At present, the most popular strategies for early identification of eclampsia are routine blood pressure readings and tests for proteinuria. Monitoring of clinical symptoms, blood tests, and ultrasound exams can also be used for detection at an early stage. However, there are restrictions on the use of these techniques. Detection may be difficult at first since early symptoms are often subtle. In addition, repeated testing and close observation are often necessary to arrive at a correct diagnosis.

Preeclampsia is a common pregnancy complication that typically occurs in the later stages of pregnancy and is characterized by symptoms such as high blood pressure and proteinuria (40). While the exact cause of pre-eclampsia is not fully understood, research suggests that mitochondria may play a role in its pathogenesis. Mitochondria are tiny structures within cells responsible for energy production and maintaining normal cellular functions. Studies have indicated that there may be abnormalities in mitochondrial function in the cells of pre-eclampsia patients (41, 42). This relationship can be explored in several ways: Firstly, Damage to cells from an excess of oxygen free radical generation occurs in pre-eclampsia patients due to oxidative stress. Oxygen free radicals are generated mostly by mitochondria. Pre-eclampsia is known to be worsened by oxidative stress, which may be further exacerbated by mitochondrial dysfunction. Second, it has been hypothesized that decreased energy production by mitochondria contributes to the symptoms of preeclampsia, which include elevated blood pressure and proteinuria. Since mitochondria are the cells’ principal source of energy, any disruption in their function could lead to a decrease in cellular energy and, in turn, the development of preeclampsia. Additionally, pre-eclampsia involves abnormalities in the immune system, including heightened immune responses. Some studies suggest that mitochondrial dysfunction may be linked to immune system abnormalities, potentially leading to irregular immune responses and the initiation of preeclampsia. In this study, we firstly analyzed GSE75010 datasets and screened 552 DE-MRGs between preeclampsia samples and normal samples. Through functional correlation analysis of 552 DE-MRGs, we have obtained a series of crucial findings regarding the pathogenesis of pre-eclampsia. Firstly, based on the results of GO analysis, these genes are primarily enriched in biological processes and molecular functions closely related to mitochondrial function, including mitochondrial transport, regulation of mitochondrial organization, and energy derivation through the oxidation of organic compounds. This strongly suggests the significant role of mitochondria in the pathological processes of preeclampsia. In addition, the KEGG analysis reveals that these genes are associated with neurodegenerative diseases such as Huntington’s disease, Parkinson’s disease, Alzheimer’s disease, as well as processes like oxidative phosphorylation and the degradation of branched-chain amino acids. This further underscores the potential regulatory role of mitochondria in neurodegenerative diseases and abnormalities in energy metabolism, which are relevant to the pathogenesis of preeclampsia. Lastly, the DO analysis indicates that these genes are related to various diseases and disease categories, including autonomic nervous system neoplasms, neuroblastomas, inherited metabolic disorders, tauopathies, Alzheimer’s disease, and muscular diseases. This suggests a possible link between mitochondrial dysfunction and the development and progression of these diseases, which also have intersections with preeclampsia. Overall, these results provide compelling evidence that mitochondrial function is important to the development of preeclampsia. Mitochondrial regulation of energy metabolism, oxidative stress, and nervous system-related pathways may all play a role in the etiology of preeclampsia. These findings have important implications for future diagnostic and therapeutic strategies, as they shed light on the pathogenic underpinnings of pre-eclampsia.

LASSO is a statistical method used for regression analysis and feature selection. It was initially introduced by the statistician Robert Tibshirani in 1996 and has found widespread application in the fields of data modeling and feature selection (43). In the context of screening diagnostic genes, LASSO is highly valuable in the domains of molecular biology and bioinformatics. When researchers need to sift through large-scale gene expression data to identify key genes associated with a specific disease or biological process, LASSO can be instrumental. By incorporating L1 regularization into linear regression models, LASSO automatically shrinks the coefficients of irrelevant or weakly related genes to zero, effectively excluding them and retaining only the genes that are relevant to the disease or biological process of interest (44). In this study, we performed LASSO analysis using 552 DE-MRGs and screened 19 critical diagnostic genes for preeclampsia. Then, we developed a novel diagnostic model using the above 19 diagnostic genes and it exhibited a strong diagnostic ability with AUC=0.950. SVM-RFE is a machine learning method primarily used for feature selection, particularly for identifying important features or genes relevant to diagnostic or classification tasks (45). It reduces the dimensionality of high-dimensional data, thereby enhancing the efficiency and generalization capability of models. In addition, by selecting the most important features, SVM-RFE significantly improves the performance of classification models, making them more discriminative and accurate. Most importantly, this process is automated and does not require subjective human intervention, helping eliminate researcher bias (46, 47). Overall, SVM-RFE is a powerful tool used for selecting genes or features relevant to diagnostic tasks. It combines the classification capabilities of Support Vector Machines with the feature selection strategy of Recursive Feature Elimination, enhancing the performance of classification models, mitigating the curse of dimensionality, and automating the feature selection process. This method finds extensive application in bioinformatics and medical research, particularly in tasks related to diagnostics, biomarker identification, and other related endeavors. In this study, we also performed SVM-RFE and screened 19 critical diagnostic genes for preeclampsia. Then, we also developed a novel diagnostic model using the 19 diagnostic genes and it exhibited a strong ability in screening preeclampsia samples from normal samples. Our findings suggested the strong ability in screening diagnostic genes.

To further optimize the diagnostic model, we integrated two machine learning algorithms and identified three crucial overlapping genes, including CPOX, DEGS1 and SH3BP5. CPOX is a gene that encodes an enzyme in the human body. This enzyme plays a crucial role in the heme biosynthesis pathway, facilitating the conversion of a precursor molecule called Coproporphyrinogen into heme (48). Heme is a critical component of hemoglobin and other hemoproteins, essential for the transport of oxygen throughout the body. Mutations or abnormalities in the CPOX gene can disrupt the heme biosynthesis pathway, potentially leading to genetic disorders such as porphyria (49). Porphyria is a group of rare inherited diseases, including subtypes like Porphyria Cutanea Tarda (PCT). PCT is one of the most common forms of porphyria and typically manifests with skin sensitivity and skin symptoms upon exposure to light. To date, the function of CPOX in preeclampsia has not been investigated. The protein encoded by the DEGS1 gene is an enzyme that plays a crucial role in lipid metabolism within the body. It is involved in regulating the synthesis of sphingolipids, which are vital lipid molecules with key functions in the nervous system (50, 51). Specifically, the DEGS1 protein participates in the conversion of dihydroceramide, a precursor molecule, into ceramide. Ceramide is a type of lipid molecule that plays essential roles in building cell membranes, maintaining cell structure, and participating in cell signaling processes. In the nervous system, particularly in the formation of myelin sheaths, ceramides are critical components for preserving the normal functioning of nerve cells and protecting nerve fibers. Therefore, the normal function of the DEGS1 gene is of paramount importance for maintaining the health of the nervous system and ensuring proper lipid metabolism. Abnormalities in the DEGS1 gene or protein may be associated with neurological disorders or other related health issues. However, the expression and function of DEGS1 preeclampsia have not been investigated. SH3BP5 is a protein that plays a crucial role in the immune system and cell signaling. It contains multiple functional domains, including the SH3 (Src homology 3) domain, which is used for protein-protein interactions (52). SH3BP5 interacts with other proteins through these domains, participating in the regulation of cell signal transduction. In the immune system, SH3BP5 plays a significant role, especially in the regulation of the activation of T cells and B cells. It plays a key role in the signaling of T cell receptors, influencing the activation and function of T cells, thereby regulating immune responses (53, 54). Abnormal expression or malfunction of SH3BP5 was associated with certain diseases and disease processes, including leukemia, lymphoma, and autoimmune diseases. Additionally, SH3BP5, through its interactions with other proteins, is involved in the regulation of multiple signaling pathways, including Ras-MAPK, PI3K-Akt, and NF-κB pathways, affecting cell survival, proliferation, differentiation, and immune responses, among other critical cellular functions. However, the function of SH3BP5 in Preeclampsia progression remained unknown. In this study, we found that the expressions of CPOX, DEGS1 and SH3BP5 were highly expressed in preeclampsia samples in GSE44711 and GSE75010 datasets and our cohort. The high expression of CPOX, DEGS1, and SH3BP5 may be associated with the pathogenesis of preeclampsia. The abnormal expression of these genes may play a role in the development of preeclampsia. Then, we developed a novel diagnostic model using CPOX, DEGS1, and SH3BP5 and it showed a strong ability in screening preeclampsia samples from normal samples. In addition, its diagnostic value was further confirmed in GSE44711 datasets and our cohorts. Our findings highlighted the potential of this new model used as a novel diagnostic biomarker. Our study established a diagnostic model based on MRGs, demonstrating excellent accuracy in the early identification of preeclampsia patients. This not only provided pregnant women with opportunities for earlier intervention but also equipped clinicians with more precise tools for managing cases of preeclampsia. From a public policy perspective, our findings may contribute to the formulation of relevant policies to ensure appropriate attention for women at higher risk of preeclampsia. This included reinforcing regular trimester screenings for pregnant women using our model, promoting broader screening, and implementing preventive measures. These initiatives have the potential to enhance the overall health of pregnant women and play a positive role in public health. Nevertheless, it was imperative to acknowledge the inherent potential for variability across distinct datasets and populations. Prudent consideration was warranted when extrapolating the findings to heterogeneous cohorts.

The immune microenvironment refers to small regions located within tissues, either inside or outside the body, where interactions among immune cells, signaling molecules, and the extracellular matrix impact the activity of the immune system (55, 56). The immune microenvironment plays a crucial role in regulating immune responses, maintaining tissue health, and responding to diseases such as infections and tumors. Within the immune microenvironment, various immune cells such as macrophages, T cells, B cells, and natural killer cells work closely together, each performing its specific immune functions. These cells communicate through the secretion and response to signaling molecules, which include cytokines, chemokines, antibodies, and inflammatory mediators (57, 58). These molecules can activate, suppress, or guide the activities of immune cells. Furthermore, the immune microenvironment also includes the extracellular matrix, which is a structural scaffold composed of proteins, polysaccharides, and other molecules. It is crucial for maintaining tissue structure and facilitating the migration and positioning of immune cells. The immune microenvironment is also associated with the inflammatory process, which is a protective immune response usually triggered by infections or tissue damage. The immune microenvironment plays a vital role in monitoring abnormal cells, including cancer cells. Immune cells can identify and eliminate these abnormal cells through cytotoxic mechanisms or other mechanisms. However, some tumors and pathogens can develop immune evasion mechanisms to escape the immune system’s attacks. In recent years, in-depth research into the immune microenvironment had driven the development of immunotherapy approaches. These treatments aim to activate the immune system to help the body combat infections, cancer, autoimmune diseases, and other conditions. Therefore, the immune microenvironment was considered a dynamic ecosystem that is crucial for maintaining immune balance and responding to various health challenges. During a normal pregnancy, the mother’s immune system undergoes a series of changes to prevent an immune response against the fetus. This immune regulation contributes to maintaining the stability and success of pregnancy (59, 60). However, in the case of preeclampsia, this immune regulation may become disrupted, leading to abnormal activation of immune cells. Inflammation and the activation of immune cells may play a crucial role in the pathogenesis of preeclampsia (61). Some studies suggested that immune cells such as T cells, macrophages, and natural killer cells may be in an abnormal state in preeclampsia patients, leading to inflammatory responses and vascular damage, thereby causing hypertension and other preeclampsia symptoms. The inflammatory process may have an important role in the development of preeclampsia. The release of inflammatory mediators and cytokines can lead to damage to vascular endothelial cells, increased vascular permeability, and the development of proteinuria and high blood pressure (62, 63). In addition, Mitochondria served as the primary source of reactive oxygen species (ROS), playing a role as signaling molecules. Controlled ROS production is crucial for normal immune responses, including the activation of immune cells. However, an imbalance in mitochondrial ROS production may lead to abnormal immune reactions at the placenta. Mitochondrial function was involved in cellular metabolism and may influence the polarization of immune cells. Metabolic changes in immune cells, triggered by mitochondrial signals, may result in immune memory. This metabolic imprint could impact long-term immune responses and tolerance at the placenta (64–66). In this study, a positive correlation was observed between the levels of SH3BP5 and specific immune cell subgroups. Specifically, SH3BP5 levels showed a positive association with CD4 naive T cells, activated dendritic cells, eosinophils, resting natural killer cells, and monocytes. This suggested that in cases of preeclampsia, elevated levels of SH3BP5 may be accompanied by an increase or activation of these immune cell subgroups. Conversely, a negative correlation was found between SH3BP5 levels and M2 macrophages, suggesting that higher levels of SH3BP5 may be associated with a reduction or suppression of M2 macrophages. These findings suggested a potential immune system dysregulation in the pathogenesis of preeclampsia. Preeclampsia is typically associated with abnormal immune activation and inflammation. However, further research is needed to gain a deeper understanding of the precise mechanisms by which SH3BP5 influenced immune cell subgroups in preeclampsia.

## Conclusion

We screened a novel diagnostic model using CPOX, DEGS1 and SH3BP5 based on Machine learning. In addition, we provided important evidences that SH3BP5 may be involved in preeclampsia progression via influencing immune microenvironment. The use of machine learning models has the potential to contribute to the development of personalized medicine because it can predict the risk of preeclampsia based on specific molecular markers in patients. This will enable healthcare providers to offer personalized care and interventions to each pregnant woman, with the aim of minimizing the risk of preeclampsia to the greatest extent possible. In summary, these findings provide new directions and opportunities for research and treatment in the field of preeclampsia. Future research and clinical practices hold the promise of further enhancing our understanding of preeclampsia and improving patient outcomes and treatment effectiveness.

## Data availability statement

The datasets presented in this study can be found in online repositories. The names of the repository/repositories and accession number(s) can be found in the article/supplementary material.

## Ethics statement

The studies involving humans were approved by the First Affiliated Hospital of Xi’an Jiaotong University. The studies were conducted in accordance with the local legislation and institutional requirements. The participants provided their written informed consent to participate in this study.

## Author contributions

PH: Conceptualization, Data curation, Investigation, Methodology, Writing – original draft, Writing – review & editing. YS: Conceptualization, Methodology, Project administration, Software, Supervision, Validation, Writing – review & editing. YY: Conceptualization, Formal analysis, Project administration, Supervision, Validation, Writing – review & editing. FB: Conceptualization, Data curation, Formal analysis, Methodology, Project administration, Visualization, Writing – review & editing. NL: Data curation, Formal analysis, Funding acquisition, Investigation, Methodology, Project administration, Writing – review & editing. DL: Data curation, Formal analysis, Investigation, Project administration, Supervision, Writing – review & editing. CL: Conceptualization, Funding acquisition, Methodology, Resources, Software, Visualization, Writing – review & editing. XL: Conceptualization, Data curation, Investigation, Methodology, Supervision, Validation, Writing – review & editing. WG: Data curation, Investigation, Methodology, Software, Supervision, Writing – review & editing. LZ: Conceptualization, Funding acquisition, Investigation, Methodology, Resources, Software, Supervision, Visualization, Writing – original draft, Writing – review & editing.
